# Seroepidemiological and biomolecular survey on *Toxoplasma gondii* in Sardinian wild boar (*Sus scrofa*)

**DOI:** 10.1016/j.fawpar.2024.e00222

**Published:** 2024-01-26

**Authors:** Maria Francesca Sini, Maria Manconi, Antonio Varcasia, Giovanna Massei, Ramona Sandu, Naunain Mehmood, Fahad Ahmed, Carlo Carta, Cinzia Cantacessi, Christian Scarano, Antonio Scala, Claudia Tamponi

**Affiliations:** a Department of Veterinary Medicine, University of Sassari, Sassari, Italy; b Botstiber Institute for Wildlife Fertility Control Europe, Department of Environment and Geography, University of York, 290 Wentworth Way, Heslington, York YO10 5NG, United Kingdom; c Department of Zoology, University of Sargodha, Sargodha, Pakistan; d School of Biomedical Sciences, University of Ulster, Coleraine, Northern Ireland, United Kingdom; e Department of Veterinary Medicine, University of Cambridge, Cambridge, United Kingdom

**Keywords:** *Toxoplasma gondii*, Foodborne disease, Zoonosis, Human infection, Host-parasite distribution, Meat consumption

## Abstract

*Toxoplasma gondii* is a zoonotic parasite able of infecting all warm-blooded animals. Toxoplasmosis is one of the major foodborne diseases globally. The consumption of wild boar (*Sus scrofa*) meat from recreational hunting has been linked to outbreaks of human toxoplasmosis. The island of Sardinia (Italy) contains a large wild boar population, thus providing an opportunity to assess the distribution of *Toxoplasma* in this species and the associated risks of transmission to humans. A total of 562 wild boars were screened: heart and meat juice samples were tested for *T. gondii* DNA via nested-PCR and IgG anti-*Toxoplasma* by commercial ELISA. Anti-*Toxoplasma* IgG were detected in 24.6% (138/562) of animals, while 37.2% (209/562) of the heart samples were PCR positive. The prevalence of *T. gondii* antibodies and DNA highlights the potential role of wild boar as an important reservoir for this parasite. The study suggests that wild boar could play a significant role in spreading the parasite to humans. As wild boar numbers are increasing throughout their range, their potential role in transmitting toxoplasmosis should be communicated to stakeholders, and the impact of different population control methods on disease transmission should be thoroughly assessed to mitigate potential threats effectively.

## 1 Introduction

Toxoplasmosis is a zoonotic parasitic disease caused by the protozoan parasite *Toxoplasma gondii* (Phylum Apicomplexa). While *T. gondii* is known to infect all warm-blooded animals, only felids act as definitive hosts and shed oocysts in their faeces. Wild boar (*Sus scrofa*) and other intermediate hosts can become infected via several routes: i) by ingesting sporulated oocysts excreted by felids that contaminate the external environment; ii) by consuming raw or undercooked meat of infected intermediate hosts containing tissue cysts or iii) congenitally via transplacental transmission. Due to its remarkable efficiency, *T. gondii* is considered the fourth most important parasite in the world (Boireau et al., 2014). One-third of the human population is estimated to be chronically infected by *T. gondii* (Almeria and Dubey, 2021). Although most infections are asymptomatic, life-threatening or fatal complications in humans can occur, particularly in immunocompromised individuals. In addition, primary infections acquired during pregnancy may result in a range of adverse outcomes, including foetal ocular infection, cranial and neurological deformities, stillbirth, and miscarriage (McCall et al., 2022). The consumption of raw and undercooked meat from farm animals is an essential source of human infection; poor hygiene practices while handling contaminated feedstuffs may further contribute to human transmission (Santoro et al., 2019). The ever increasing numbers of wild boar worldwide, and particularly in Europe (Massei et al., 2015; Jori et al., 2021), as well as the growing popularity of wild boar meat in different parts of the world (Rostami et al., 2017), underpins the increased risk of toxoplasmosis transmission through consumption of meat derived from recreational hunting. Indeed, over the last few years, epidemiological investigations have been conducted worldwide to assess the role of wild boar in the transmission of *T. gondii* (Rostami et al., 2017; Olsen et al., 2019). In Europe, *T. gondii* seroprevalence in wild boar is highly variable both within and between countries, ranging from 10 to 50% (Gazzonis et al., 2018). Moreover, in several European countries, limited data are available on prevalence of toxoplasmosis in wild boar populations. In Mediterranean islands, a single epidemiological survey on wild boar has been carried out in Corsica (Richomme et al., 2010). In Sardinia, prevalence for *T. gondii* in pigs and sheep is 54.5% (Pipia et al., 2018), and 77.5% (Dessì et al., 2022), respectively. Nevertheless, thus far, no information on wild boar is available. Therefore, the present study aimed to investigate the presence of *T. gondii* in wild boar in Sardinia using biomolecular and serological techniques, and to discuss the findings in relation to potential risks for human infection.

## 2 Materials and methods

The sample size for this survey (*n* = 377) was calculated a priori using the Raosoft Digital Sample Size Calculator (RaoSoft, Inc., Seattle, WA; http://www.raosoft.com/samplesize.html), with 5% margin of error, and a 95% confidence interval (CI). A population size of 18,750 wild boar was determined based on the data provided by the Carta delle Vocazioni Faunistiche della Sardegna (Apollonio et al., 2011). A prevalence rate of 50% was considered. Wild boar examined in the present study (*n* = 562) originated from the hunting seasons of 2021–2022 throughout Sardinia (40°N 9°E) (23,812, 6 km^2^). Animal data were recorded according to age range, gender and municipality of origin, and the carcass examination was performed by veterinaries during routine post mortem surveillance procedures for the control of African Swine Fever and trichinellosis. The age of each animal was estimated by patterns of tooth eruption and replacement according to Massei and Toso (1993). Adults were defined as animals of ≥12 months-old while juveniles were defined as <12 months old. For five (n = 5) samples, age and gender were not recorded but these samples were included in the study; however, they were not included in the risk factor analysis (see below). Organs and viscera from abdominal and thoracic cavities were inspected and removed from each carcass and the hearts sent for parasitological examination to the Parasitology Laboratory of the Department of Veterinary Medicine, University of Sassari, Italy. Upon arrival, the hearts were portioned into 50 g aliquots and stored at −20 °C in a plastic bag to collect the heart meat juice for subsequent serological analysis. The heart meat juice was collected after thawing each bag containing samples at room temperature and then transferred into 1.5 ml Eppendorf microtubes (Nöckler et al., 2005). Heart tissue samples were homogenized using an Ultra Terrex® homogenizer (IKA, Stiffen, Germany). To avoid DNA cross contamination between samples, all devices were washed several times with sodium hypochlorite solution (2.5%) followed by distilled water (Santos et al., 2010). After homogenization, aliquots of 25 mg of each sample were stored at −20 °C for subsequent biomolecular examination. DNA was extracted from 25 mg of homogenized heart tissues using a commercial kit (G-spins total DNA extraction kit, Korea), according to the manufacturer's instructions. *Toxoplasma gondii* DNA was detected through a Nested PCR, as described by Pipia et al. (2018). Briefly, 302 base pairs (bp) – fragment of the Internal Transcribed Spacers-1 (ITS1) region of 18S-5,8S rRNA was amplified, according to Halová et al. (2013). Each PCR reaction was conducted in a final volume of 25 μl containing PCR buffer (10×), MgCl2 (1.0 mm), deoxynucleotide triphosphate (dNTPs) (0.5 mm of each), primer FOR (0.5 μm), primer REV (0.5 μm), 0,75 U Taq polymerase (Biotechrabbit GmbH, Berlin Germany). Positive and negative controls were included in each PCR reaction. As reference material, *T. gondii* genomic DNA was obtained from *T. gondii* positive swine heart sample from a previous study conducted in the same laboratory by Pipia et al. (2018). After extraction, DNA concentration was determined using a NanoDrop© Lite spectrophotometer. Additionally, internal extraction was conducted. PCR amplification products were resolved in 2% agarose gel and visualized by UVIdoc HD2 (UVITEC, Cambridge, UK). Fragment length estimation was carried out using the Thermo Scientific GeneRuler molecular weight standard (100 bp DNA ladder). PCR-positive samples were purified using nucleospin gel and PCR clean up (Macherey-Nagel GmbH & Co. KG, Düren, Germany) and sequenced by a commercial service (Eurofins Genomics, Ebersberg, Germany) in order to confirm the specificity of the PCR amplifications. The sequences obtained were compared with those available in the National Centre for Biotechnology Information (NCBI) database using the Basic Local Alignment Search Tool (BLAST) (http://www.ncbi.nlm.nih.gov/BLAST/).

The heart meat juice from each sample was tested for the presence of anti-*Toxoplasma* IgG using a commercial enzyme linked immuno-adsorbent (ELISA) kit (PrioCHECK® *Toxoplasma* Ab SR, Prionics, Schlieren-Zurich, Switzerland), as previously described (Dessì et al., 2022). All animal data, along with the results of PCR and ELISA, were recorded on a spreadsheet (Microsoft Excel®, Microsoft Corp., Redmond, WA). The prevalence at the individual level was computed with an associated confidence interval of 95% (95% CI). Data were subsequently analysed using *Chi*-square test (χ^2^) (Epi-info 6.04, CDC, Atlanta, GA, USA), and results were considered statistically significant if *P* < 0.05.

## 3 Results

The present study revealed an overall prevalence of 37.1% for *T. gondii* (209/562; CI95%: 33.2–41.2) detected by PCR. Female boar yielded a prevalence of 39.7% (106/267; CI95%: 33.8–45.6), while males of 34.8% (101/290; CI95%: 29.3–40.3), albeit no statistically significant difference in infection prevalence was recorded between genders (χ2 = 1.41; *P* = 0.23). A prevalence of 39.4% (158/401; CI95%: 34.6–44.2) and 31.4% (49/156; CI95%: 24.1–38.7) was detected in adults and juveniles, respectively, although this difference was also not statistically significant (χ2 = 3.07; *P* = 0.079). Of the5 samples for which gender and age of wild boar had not been recorded 2 tested positive by PCR. Nested PCR results were confirmed by sequencing of representative samples, which displayed 99% homology to *T. gondii* sequences available in GenBank (accession number JX456457.1) (Table 1).

**Table 1:** Number of positive cases, prevalence of *Toxoplasma* antibodies and PCR results divided per age (adult ≥12 months, juveniles <12 months) and gender.

Animal category	Examined wild boar	Number of Positive Samples		Prevalence		CI (95%)	
		**ELISA**	**PCR**	**ELISA**	**PCR**	**ELISA**	**PCR**
**Male**	290	84	101	29.0%^a^	34.8%^c^	23.7–34.2	33.8–45.6
**Female**	267	51	106	19.1%^a^	39.7%^c^	14.4–23.8	29.3–40.3
**Adult**	401	112	158	27.9%^b^	39.4%^d^	25.3–34.1	34.6–44.2
**Juveniles**	156	23	49	14.7%^b^	31.4%^d^	9.2–20.3	24.1–38.7

CI 95% confidence interval; ^a^χ2 = 7.366; P = 0.006); ^b^χ2 = 10.635; P = 0.001; ^c^χ2 = 1.41; P value = 0.23; ^d^χ2 = 3.07; *P* value = 0.079.

An overall prevalence of 24.6% (138/562; CI95%: 21.0–28.1) for anti-*Toxoplasma* IgG was detected in samples subjected to ELISA; in particular, a significantly higher prevalence (29.0%; 84/290; CI95%: 23.7–34.2) was detected in males than in females (i.e. 19.1%; 51/267; CI95%: 14.4–23.8) (χ2 = 7.366; *P* = 0.006). Likewise, the adult cohort (i.e. 27.9%; 112/401; CI95%: 25.3–34.1) displayed significantly higher seroprevalence than juveniles (i.e. 14.7%; 23/156; CI95%: 9.2–20.3) (χ2 = 10.635; *P* = 0.001) (Table 1). Three out of the 5 unidentified samples were positive for IgG antibody *T. gondii.*

## 4 Discussion

The present survey represents the first comprehensive epidemiological study of *T. gondii* in wild boar in a large Mediterranean island. The sample size in this study (*n* = 562) exceeded the minimum number of 377 samples initially required, thus strengthening the robustness of the experimental design. The 37.1% prevalence of *T. gondii* recorded in the wild boar population indicates a widespread occurrence of the parasite throughout the island. Nevertheless, the *T. gondii* prevalence recorded by PCR is lower than that detected in other studies conducted in Southern Italy by Santoro et al. (2019) (44%) and Sgroi et al. (2020) (39.6%), but higher than that reported in Northern Italy (16%) (Ferroglio et al., 2014). Seroprevalence rates of *T. gondii* in wild boar vary considerably throughout the globe, i.e. 22.7% in Japan (Saito et al., 2021), 36% in Korea (Jeong et al., 2014), 27.7% in the United States (Sandfoss et al., 2011), 12.5% in Argentina (Winter et al., 2019) and 15.6% Brazil (Brandão et al., 2019). In Europe, high seroprevalence rate have been reported in Slovenia (62%) (Bandelj et al., 2021), Poland (48%) (Puchalska et al., 2021), Sweden (50%) (Wallander et al., 2015), the Czech Republic (40%) (Račka et al., 2015), Romania (56.6%) (Grema et al., 2015), and Corsica (55%) (Richomme et al., 2010). Conversely, relatively low seroprevalence rates have been recorded in Slovakia (8.1%) (Antolová et al., 2007) and Switzerland (6.7%) (Berger-Schoch et al., 2011). A PCR study conducted in the Czech Republic showed a low prevalence (8.8%) while a higher seroprevalence rate (15.4%) was recorded (Slany et al., 2016), likely attributed to the use of diaphragmatic instead of cardiac tissue. The overall seroprevalence (24.5%) recorded in our study is similar to that reported in Germany (24.40%) (Bier et al., 2020), and in the Netherlands (24.4%) (Opsteegh et al., 2011), but higher than those recorded in Spain (14%) (Lizana et al., 2021) and Central Italy (14%) (Ranucci et al., 2013). These variations emphasize the challenges with comparing seroprevalences among different surveys, which are conducted under different circumstances in geographically distinct areas, and through the application of different diagnostic techniques and epidemiological approaches (Veronesi et al., 2011). It is essential to note that this study used meat juice as a serological matrix, which differs from blood serum. ELISAs applied to this material are characterised by lower sensitivity than blood serum. However, specific antibody levels in meat juice vary depending on the muscle(s) sample, with heart meat juice characterised by significantly higher levels of antibodies compared with other muscles (Wallander et al., 2015). ELISA can only identify chronic infections via the detection of anti-*Toxoplasma* IgG, while PCR analysis detects nucleic acids from *T. gondii* (Dessì et al., 2022). Importantly, previous studies have shown that the detection of *T. gondii* antibodies in wild boar is positively correlated with the presence of bradyzoites in the animal muscles (Bártová et al., 2006; Richomme et al., 2010).

In our survey, animal gender and age were identified as risk factors for *T. gondii* infection, with significantly higher seroprevalences recorded in males (29.0%) compared to females (19.1%) and in adults (27.9%) compared with juveniles (14.7%). The higher seroprevalence in male wild boar is not consistent with data from other studies, that reported no gender-related difference in seroprevalence (Richomme et al., 2010; Opsteegh et al., 2011; Ranucci et al., 2013). This may be explained by the larger home ranges of males compared to females (Laguna et al., 2022; Cavazza et al., 2023).The higher prevalence observed in adult wild boar is also not consistent with data from other studies and it may be related to longer exposure to the parasite compared with juveniles (Antolová et al., 2007; Opsteegh et al., 2011; Ranucci et al., 2013). However, whether anti-*T. gondii* immunity in wild boar is a lifelong condition remains to be determined. Opsteegh et al. (2011) showed that animals up to 10 months of age display a step-increase in seroprevalence, with titres of anti-*Toxoplasma* antibodies stabilising thereafter. This suggest that susceptibility to infection may remain throughout an animal's life. Based on our PCR data, gender and age likely to represent a risk factors, with a higher PCR prevalence in females (39.7%) than males (34.8%) and in young animals (39.4%) compared to adults (31.2%), although differences between categories are minimal. The stress associated with pregnancy and breastfeeding in females may lead to an increased susceptibility to *T. gondii* infection, a hypothesis that requires testing. Similarly, the higher prevalence recorded in young wild boar may be linked to instances of transplacental transmission and/or the developing immune system (Dubey et al., 1990; Pipia et al., 2018). However, this data contrast with widespread knowledge that *T. gondii* prevalence rates are higher among older animals compared to piglets. This discrepancy is usually attributed to the higher likelihood of older wild boars coming into contact with infective oocysts or infected intermediate hosts (Herrero et al., 2016).

Our data support the hypothesis that infected wild boar may represent a key-sentinel of environmental contamination with *T. gondii*, due to their capacity for adaptation to different habitats, wide geographical distribution and high reproductive rates (Sgroi et al., 2020). Recreational hunting can impact wild boar social and spatial behaviour (Keuling and Massei, 2021). For instance, a study in Catalonia (Spain) found six of 40 wild boar, which had been ear-tagged and later culled by hunters, at a mean linear distance of 45.8 km (min. 30, max. 89.8) from their origin (Casas-Díaz et al., 2013). Wild boar could play an important role in maintaining the *T. gondii* sylvatic cycle due to their scavenging and predatory behaviour, and frequent interactions with a wide range of different hosts (including sheep, cattle, birds, rodents and foxes).

Hunter malpractices, such as inconsiderate disposal of animal offal in the environment following evisceration, could exacerbate the perpetuation of *Toxoplasma* life cycle (Sgroi et al., 2019). Wildlife animals, like wild boar, predators (including cats) or pigs, can scavenge offal and viscera, indirectly amplifying transmission risks within the food chain and, consequently, the risk of human exposure (Ranucci et al., 2013). The detection of *T. gondii* in wild boar meat highlights a potential risk to humans via the growing market of wild boar sausages and cured meat (Richomme et al., 2010). These latter products have previously been shown a risk for infection with pathogenic microorganisms, including *T. gondii* (Hill and Dubey, 2018; Fredericks et al., 2019). Sausages and fresh meat processed through smoking, salting, drying, and injection of solutions containing sodium chloride, potassium lactate and sodium lactate are intended for direct consumption. However, some of these techniques may inactivate bradyzoites pathogen transmission remains possible (Hill et al., 2006; Kijlstra and Jongert, 2008; Hill and Dubey, 2018). In particular, the “Salsiccia Sarda” (Sardinian sausage), a short-aged, cured salami, made with meat and fat from pork, is a traditional Sardinian product included in the national list of traditional agri-food products (XXIII Revision of the list of traditional agri-food products produced, Italian Republic, 22.05.2023). Curing for >12 months is recommended to ensure the inactivation of *T. gondii* in many salami (Fredericks et al., 2019). Nevertheless, for the Sardinian sausage, this time is reduced to a maximum of 20–25 days, potentially allowing any *T. gondii* cysts to maintain their infectious properties throughout the curing process.

The ever-rising number of wild boar in many countries, Sardinia included, has led hunters to consume wild boar game meat by also producing fresh sausages, thus increasing the risk of transmission to humans. Consumption of raw or undercooked wild boar meat may lead to infection, as evidenced by several reported cases of acute toxoplasmosis among hunters and their families who consumed meat from infected wild boar (Choi et al., 1997). It is essential to point out that simple and inexpensive methods, such as heating, cooking, and freezing, are effective in inactivating *Toxoplasma* cysts. Several studies have demonstrated that heating water to 60 °C for 1 min can efficiently inactivate tachyzoites. Additionally, cooking meat at 67 °C is effective in killing the parasite. Freezing meat for at least three days at −20 °C reduces the *T. gondii* load in contaminated meat. Cross-contamination between raw and processed food products must nevertheless be prevented (Mirza Alizadeh et al., 2018). Consequently, hunters can also become infected during evisceration practices and at various stages of venison processing (Tenter et al., 2001).

The prevalence of *T. gondii* in pigs, particularly those intended for family consumption, is higher than that in wild boar, as shown by Pipia et al. (2018) in Sardinian organic pig farms (85.7% and 54.8% seroprevalence and PCR, respectively). This could be attributed to several factors, including high environmental contamination, occurrence of wild and/or stray cats around the farms, the practice of raising domestic pigs alongside other animals (sheep, goats, or cattle) and the persistence of a sylvatic cycle, e.g. though wild boar. Given the widespread consumption and easy access to pig meat, this aspect merits further consideration.

To address this concern, the Biological Hazard Panel of the EFSA has recently proposed the implementation of *Toxoplasma* monitoring programmes (EFSA, 2007). These programs aim to produce *Toxoplasma*-free meat via selection of seronegative animals and the introduction of validated tests and certification, leading to the issuance of ‘*Toxoplasma*-free meat’ label for *Toxoplasma*-free farms (Kijlstra and Jongert, 2008). Furthermore, the development and standardization of methods for testing of pathogen survival in dry-cured meat products may assist with mitigating the risk of *Toxoplasma* transmission to consumers (Herrero et al., 2016). At present, Italian law does not enforce health checks on meat obtained from hunted wild boar. Biological samples from hunted wild boar are typically tested for the presence of *African Swine Fever* (ASF) and *Trichinella* spp. (according to the European Regulation 2015/1375), which outlines rules for *Trichinella* control in meat and no specific checks are required for other pathogens. However, this game meat is frequently offered in typical country restaurants (“agritourism”) to locals and tourists alike, who often consume raw homemade products (e.g., sausages or “guanciale”). Due to this potential risk to public health, these measures could be adopted to reduce the potential role of wild boar in the zoonotic transmission of *T. gondii*, alongside an educational effort to raise public health awareness.

## 5 Conclusions

This study underscores the high prevalence of *T. gondii* in Sardinia's wild boar population that poses a substantial risk of transmission to humans. To address this concern, it is recommended for regulatory agencies to implement comprehensive control programmes. These may include public education, implementation of specific regulatory legislations to adopt methods of wild boar density reduction (e.g. through controlled hunting or fertility control programmes) and monitoring of the whole food chain. Subsequently, the adoption of specific sanitary measures and training courses focusing on appropriate application of safe hygiene practices among hunters, such as contamination prevention, cooking and freezing, are pivotal to improve and ultimately guarantee food safety.

## Declaration of competing interest

The authors declare that they have no known competing financial interests or personal relationships that could have appeared to influence the work reported in this paper.
